# The Role of miR‐138 in Cardiovascular Diseases

**DOI:** 10.1155/bmri/2356842

**Published:** 2025-12-22

**Authors:** Taidou Jiang, Bijian Wang, Zhi Luo, Ying Xia, Yaoyu Qi, Sha Luo, Binyan Lang, Bolan Zhang, Shuzhan Zheng

**Affiliations:** ^1^ Department of Cardiology, The People′s Hospital of Jianyang City, Jianyang, China; ^2^ Department of Cardiology, Leshan People′s Hospital, Leshan, China; ^3^ Department of Cardiology, Affiliated Hospital of Southwest Medical University, Luzhou, China, ahswmu.cn

**Keywords:** atherosclerosis, cardiovascular diseases, miR-138, miRNA, ischemia reperfusion injury, heart failure, pulmonary arterial hypertension

## Abstract

Cardiovascular disease (CVD) represents the foremost cause of morbidity and mortality globally, posing a significant threat to human health. The regulatory mechanisms underlying CVD are still not fully elucidated. MicroRNA (miRNA), a class of noncoding short‐chain RNA molecules, modulates individual genes or gene networks by binding to the complementary sequences of specific target genes, thereby influencing various biological processes including cell genesis, metabolism, proliferation, differentiation, and apoptosis. Among these, miR‐138 plays a significant role in the onset and progression of CVDs. This article reviews the involvement of miR‐138 in various cardiovascular conditions, including atherosclerosis (AS), myocardial ischemia–reperfusion injury (I/R), heart failure (HF), and pulmonary arterial hypertension (PAH), thereby offering novel insights for the prevention, diagnosis, and treatment of CVDs.

## 1. Introduction

Cardiovascular diseases (CVDs) represent the predominant cause of global morbidity and mortality [1]. While hypertension, diabetes, hypoxia, and advanced age constitute established risk factors for CVD pathogenesis, their underlying regulatory mechanisms remain incompletely characterized. MicroRNAs (miRNAs) constitute a class of endogenous noncoding small RNA molecules that bind complementary sequences of target mRNAs, mediating posttranscriptional gene silencing through mRNA degradation or translational repression. These molecules regulate fundamental cellular processes including proliferation, metabolism, differentiation, and apoptosis [2]. Accumulating evidence demonstrates that multiple miRNAs orchestrate critical pathways in CVD pathogenesis, modulating myocardial ischemia, hypertrophic remodeling, fibrotic progression, angiogenic regulation, dyslipidemia, arrhythmogenesis, and heart failure (HF) development [3]. Circulating miRNA dysregulation (e.g., miR‐133a, miR‐21, and miR‐499) in serum/plasma exhibits diagnostic, prognostic, and risk stratification utility for clinical entities including acute coronary syndromes and HF [4]. Research into miRNAs is fundamentally reshaping CVD diagnostics and therapeutics. Within this landscape, miR‐138 has emerged as a molecule of significant interest due to its role as a master regulator of key cardiovascular processes. These include maintaining vascular homeostasis, as well as modulating pathological processes such as atherosclerotic plaque progression, vascular calcification, myocardial remodeling, and the pathogenesis of metabolic cardiomyopathy. The pleiotropic nature of miR‐138′s actions, its position as a central regulatory node, and its substantial translational potential distinguish it from other miRNAs and designate it as a high‐priority research target. This review synthesizes current mechanistic insights into miR‐138 and its clinical implications for CVD, thereby laying the groundwork for innovative strategies in prevention, diagnosis, and treatment.

## 2. Molecular Characteristics, Regulation, and Therapeutic Delivery of miR‐138

### 2.1. Basic Characteristics of miR‐138

miRNAs are transcribed by RNA polymerase II to generate primary transcripts (pri‐miRNAs). These pri‐miRNAs are subsequently processed in the nucleus into precursor miRNAs (pre‐miRNAs), which are characterized by a stem‐loop structure typically 70 nucleotides in length. This precursor is then cleaved by the ribonuclease enzyme Dicer in the cytoplasm, yielding two products: the miRNA that matures near the 5 ^′^ stem, referred to as miR‐5p, and the one maturing from the 3 ^′^ end, designated as miR‐3p [5]. miR‐138 was first identified by Lagos‐Quintana in the mouse cerebral cortex. Its mature sequence is 5 ^′^‐AGCUGGUGUUGUGAAUC‐3 ^′^ [6]. As a member of the miRNA family, miR‐138 comprises two paralogs, miR‐138‐1 and miR‐138‐2, which map to human chromosomes 3 (Ch3p21.32) and 16 (Ch16q13), respectively [7]. While pre‐miR‐138‐2, the precursor of miR‐138, is widely expressed across multiple tissues, mature miR‐138 shows tissue‐ and cell type–specific expression patterns. Following nuclear processing, pre‐miR‐138‐2 is transported to the cytoplasm, where it is cleaved by the ribonuclease Dicer to generate the mature form, designated hsa‐miR‐138‐5p, which then exerts its biological functions [8]. Mature miR‐138 is expressed in diverse cell types and tissues, including brain, heart, liver, lung, kidney, and the gastrointestinal system. It plays pivotal roles in fundamental cellular processes, such as proliferation, differentiation, apoptosis, migration, and invasion. Dysregulation of miR‐138 contributes to the pathogenesis and progression of multiple diseases, including CVD, cancers, and neurological disorders [9–11]. Critically, miR‐138 plays an essential role in cardiac development and is indispensable for proper heart formation. Furthermore, altered miR‐138 expression levels in cardiovascular tissues correlate strongly with the progression and severity of various CVDs. Therefore, miR‐138 represents both a significant biomarker and a promising therapeutic target for CVD.

### 2.2. Regulatory Mechanisms of miR‐138

The activity of miR‐138 is tightly regulated at multiple levels. A major mechanism involves competing endogenous RNAs (ceRNAs), including long noncoding RNAs (lncRNAs) and circular RNAs (circRNAs), which function as molecular sponges to sequester miR‐138‐5p and reduce its bioavailability [12, 13]. This sponging activity results in derepression of miR‐138 target genes and contributes to diverse pathological processes. For example, circ‐FAM158A sponges miR‐138‐5p to promote retinoblastoma progression by upregulating SLC7A5 [14], while circ_0082476 sequesters miR‐138‐5p and enhances BRD4 expression, thereby facilitating proliferation, migration, and inflammation in keratinocytes [15]. In the cardiovascular field, circ‐CCND1 functions as a ceRNA in valve interstitial cells, sponging miR‐138‐5p and driving aortic valve calcification. The testis‐specific circRNA, Sry, was also identified as an early miR‐138 sponge [12, 16]. Similarly, lncRNAs including Linc‐ROR, H19, and KCNQ1OT1 regulate osteogenesis and vascular calcification by suppressing miR‐138 activity [17, 18].

Beyond RNA sponging, miR‐138 is also subject to transcriptional and epigenetic regulation. The transcription factor HES1 inhibits the transcription of both miR‐138‐5p and miR‐138‐2‐3p by binding directly to the miR‐138‐2 promoter, thereby sustaining NOTCH1 signaling in renal cell carcinoma [19]. Epigenetic regulators also play important roles; for instance, EZH2 represses miR‐138 by inducing promoter methylation in osteoarthritis [14], while TGF‐*β* signaling is thought to downregulate miR‐138 during the pathogenesis of systemic sclerosis [20]. Together, these findings underscore the complexity of miR‐138 regulation and highlight the importance of ncRNA networks and epigenetic factors in modulating its function in cardiovascular and noncardiovascular contexts.

### 2.3. Pharmacological Delivery Strategies of miR‐138

#### 2.3.1. Synthetic Agents

Pharmacological modulation of miR‐138 primarily relies on synthetic agents, including mimics and inhibitors. miR‐138 mimics are designed to restore or enhance its activity, thereby suppressing cell proliferation and promoting apoptosis in breast cancer and glioma models [21, 22]. Importantly, systemic administration of miR‐138 mimics has been shown to inhibit vitamin D3–induced aortic valve calcification in vivo, highlighting their therapeutic potential in CVD [13]. Conversely, miR‐138 inhibitors (antagomirs) reduce its activity and have shown promise in skeletal disorders [23]. Inhibition of miR‐138 enhances bone formation and mechanical strength in aged mice, prevents bone loss under mechanical unloading, suppresses apoptosis in ovariectomy‐induced osteoporosis [13], and alleviates inflammation while promoting wound healing in diabetic foot ulcer models [24].

#### 2.3.2. Delivery Systems

The effectiveness of these therapeutic approaches depends critically on the delivery systems employed. For localized orthopedic applications, chitosan–miR‐138 inhibitor complexes embedded in titanium screws have been implanted into rat bones to enhance osteointegration [19]. In the central nervous system, a sophisticated strategy leverages engineered microglia carriers: Microglia loaded with miR‐138‐5p via glycosylation engineering and bioorthogonal click reactions exploit their inflammatory chemotaxis to deliver miR‐138 directly to ischemic regions, protecting hypoxic neurons by enhancing mitophagy [23]. In addition, standard nonviral vectors such as Lipofectamine2000 remain widely used for in vitro delivery of mimics and inhibitors across multiple cell types, including human aortic valve interstitial cells (AVICs) [25]. Collectively, these delivery methods demonstrate the pharmacological feasibility of targeting miR‐138 in cardiovascular as well as other disease contexts.

## 3. Relationship Between miR‐138 and CVDs

### 3.1. Atherosclerosis (AS)

AS is the pathological basis of CVDs such as coronary heart disease. In the early stage of AS, dysfunctional endothelial cells recruit circulating monocytes into the subendothelium, where they differentiate into macrophages. These macrophages engulf lipids to form foam cells and secrete inflammatory factors that amplify vascular inflammation. Meanwhile, smooth muscle cells migrate and proliferate in the subendothelium, eventually leading to plaque formation [26]. Increasing evidence indicates that miRNAs play pivotal roles in cholesterol metabolism, cell adhesion, inflammation, and smooth muscle cell proliferation [27]. Among them, miR‐138 is closely linked to AS pathogenesis, particularly through endothelial dysfunction, smooth muscle cell proliferation, and inflammatory regulation.

As a member of the miRNA family, miR‐138 is intricately associated with the pathogenesis of AS, particularly affecting endothelial cell dysfunction, smooth muscle cell proliferation, and inflammatory processes. This process involves multilayered pathological mechanisms: (1) Disruption of endothelial homeostasis: Reduced nitric oxide (NO) production catalyzed by endothelial nitric oxide synthase (eNOS) leads to impaired vasodilation and anti‐inflammatory functions [28]; (2) Amplification of inflammatory cascades: Foam cells release proinflammatory cytokines that activate signaling pathways such as nuclear factor kappa‐B (NF‐*κ*B), thereby perpetuating a vicious cycle of inflammation; and (3) Dysregulation of signaling pathways: Imbalanced activity within the Sirtuin 1 (SIRT1), APT1/H‐Ras/MAPK, PI3K/AKT, and other pathways accelerates atherosclerotic progression [29–33]. Endothelial cells are rich in endothelial nitric eNOS, which catalyzes the production of NO from the substrate L‐arginine (L‐Arg). When the amount of NO produced by this pathway decreases, it will lead to abnormal endothelial cell function, resulting in a series of cardiovascular system dysfunctions, which may ultimately lead to the development of AS [28].

Detailed mechanistic studies have demonstrated that miR‐138 negatively regulates eNOS activity by targeting S100A1, a Ca2+ sensor critical for eNOS activation. Hypoxia‐induced miR‐138 overexpression binds to the 3 ^′^UTR of S100A1, leading to eNOS inactivation, decreased NO production, and endothelial dysfunction [34]. Proinflammatory factors such as angiotensin II, endothelin, and tumor necrosis factor can also induce the expression of miR‐138, thereby downregulating the expression of S100A1 and reducing NO production [35]. Furthermore, flavonoids such as safflower yellow B (SYB) effectively downregulate miR‐138 expression, upregulating S100A1 levels, thereby enhancing eNOS activity and mitigating endothelial cell damage caused by oxygen glucose deprivation (OGD) [36].

In parallel, miR‐138 modulates the activity of SIRTs, NAD‐dependent deacetylases with broad roles in vascular biology [37]. SIRT1 in particular suppresses vascular smooth muscle cell (VSMC) proliferation and vascular inflammation [38]. Xu et al. reported that miR‐138 promotes VSMC proliferation and migration in Type 2 diabetic mice by inhibiting SIRT1 expression [39]. Furthermore, downregulation of SIRT1 enhances inflammatory cytokine release, including MCP‐1, TNF‐*α*, IL‐6, and IL‐1*β* [29, 30]. Bai et al. further demonstrated that miR‐138 directly binds to SIRT1, suppresses its activity, and promotes macrophage inflammation through AKT inhibition and NF‐*κ*B activation [31].

Nevertheless, miR‐138 may also exert protective effects under specific conditions. Li et al. showed that in a human coronary endothelial cell (HCAEC) injury model, miR‐138 upregulation suppressed the PI3K/AKT/eNOS pathway, reduced proinflammatory cytokines (TNF‐*α*, IL‐6, and IL‐8), and attenuated endothelial dysfunction [32]. In addition, miR‐138 overexpression was found to inhibit the APT1/H‐Ras signaling pathway, suppress downstream MEK/ERK activation, and reduce expression of MMP‐9, VCAM‐1, and ICAM‐1. These effects delayed AS progression in ox‐LDL‐induced endothelial dysfunction and in ApoE−/− mice on a high‐fat diet [33].

**Figure 1:**
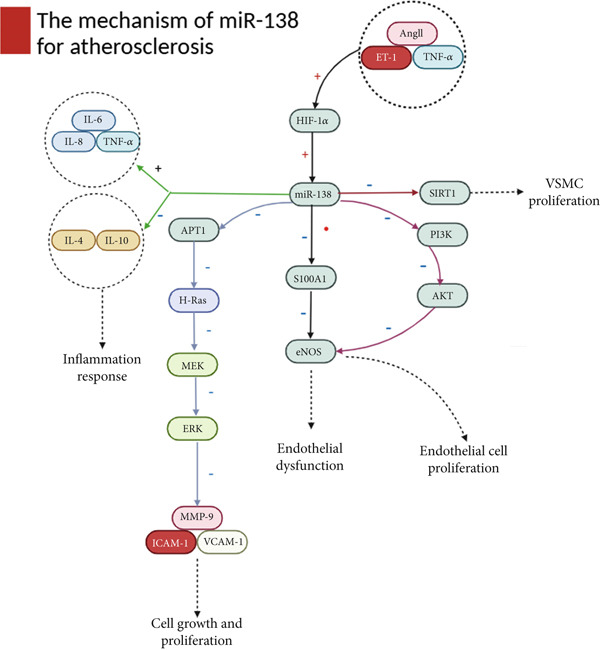
Proinflammatory factors, including angiotensin II, endothelin, and tumor necrosis factor, can induce the expression of miR‐138, downregulate S100A1 expression, inactivate eNOS, and contribute to endothelial cell dysfunction. Furthermore, miR‐138 promotes VSMC proliferation and migration by inhibiting SIRT1 expression. This mechanism is implicated in the pathogenesis of AS. Conversely, miR‐138 can inhibit the PI3K/AKT/eNOS signaling pathway, thereby reducing the expression of inflammatory factors such as TNF‐*α*, IL‐6, and IL‐8 while mitigating HCAEC damage and inflammatory responses. Additionally, miR‐138 overexpression inhibits the APT1/H‐Ras signaling pathway, subsequently suppressing MEK/ERK activation and downstream expression of AS‐related factors MMP‐9, VCAM‐1, and ICAM‐1; this ultimately delays the progression of AS.

Taken together, miR‐138 exerts dual, context‐dependent effects in AS. While it promotes disease progression by impairing endothelial function, enhancing VSMC proliferation, and amplifying inflammation, it can also protect against vascular injury by modulating PI3K/AKT/eNOS and APT1/H‐Ras/MAPK signaling. Its overall impact therefore depends on the molecular targets and disease context (Figure 1).

### 3.2. Ischemia–Reperfusion (I/R) Injury

**Figure 2:**
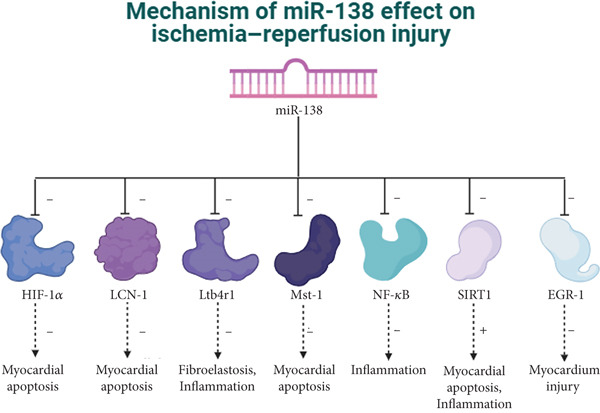
miR‐138 can reduce myocardial cell apoptosis by inhibiting the expression of downstream targets HIF‐1*α*, LCN2, and Mst‐1, as well as by suppressing the Ltb4r1 and NF‐*κ*B signaling pathways to mitigate inflammatory responses. Additionally, miR‐138 protects cardiomyocytes from hypoxia‐induced damage through the inhibition of EGR‐1. Conversely, during myocardial infarction, miR‐138‐5p may exacerbate cardiomyocyte apoptosis and inflammation by downregulating SIRT1 expression.

Acute myocardial infarction (AMI), a severe manifestation of coronary artery disease, results from abrupt reduction or cessation of coronary blood supply. As the standard therapeutic intervention, reperfusion therapy effectively salvages ischemic myocardium and limits infarct size. However, AMI can trigger myocardial I/R injury, which promotes adverse ventricular remodeling postinfarction—a major determinant of chronic HF and mortality among AMI patients [40]. The pathophysiological mechanisms of I/R injury involve oxidative stress (OS), intracellular calcium overload, vascular endothelial dysfunction, endoplasmic reticulum stress, and sustained inflammatory responses [41]. In this pathological context, miR‐138 has emerged as a protective regulator, alleviating myocardial injury by modulating multiple molecules and signaling pathways, such as HIF‐1*α*, LCN2, LTB4R1, lncRNAs, circRNAs, and EGR1 (Figure 2).

One of the earliest identified targets is hypoxia‐inducible factor 1*α* (HIF‐1*α*), which is upregulated during I/R and generally exerts cardioprotective effects by reducing ROS release and apoptosis [42]. Interestingly, miR‐138 mitigates I/R‐induced damage via HIF‐1*α* inhibition, thereby improving mitochondrial function and reducing cardiomyocyte apoptosis [43]. Consistently, in primary cardiomyocytes exposed to hypoxia/reoxygenation (H/R), astaxanthin (AST) elevated miR‐138 expression, suppressed HIF‐1*α*, and attenuated Caspase‐9 and Caspase‐3 activation, ultimately protecting against myocardial I/R injury [44].

Lipocalin‐2 (LCN2), a secreted adipokine associated with lipid binding, participates in cell survival, migration, and differentiation. Elevated circulating LCN2 correlates with coronary artery disease severity and contributes to granulocyte infiltration and chemokine production in AMI [45]. Xiong et al. demonstrated that miR‐138 prevents hypoxia‐induced cardiomyocyte apoptosis by suppressing LCN2 expression [46]. Similarly, leukotriene B4 (LTB4), an inflammatory lipid mediator, is implicated in I/R pathology. While LTB4 receptor antagonists were shown to protect against I/R in mice [47], Chang et al. reported that dexmedetomidine attenuates myocardial injury and collagen deposition in a mouse model of LAD ligation by upregulating miR‐138‐5p and downregulating LTB4R1, thereby suppressing inflammation [48].

Beyond direct molecular targets, miR‐138 interacts with noncoding RNAs that act as upstream regulators in I/R injury. lncRNAs, transcripts > 200 nt in length, are increasingly recognized as modulators of I/R‐related pathologies across multiple organs [49–52] and may serve as diagnostic biomarkers [53]. Among them, lncRNA ROR aggravates cardiomyocyte pyroptosis, inflammation, and OS during I/R, whereas miR‐138 overexpression protects H9C2 cells against ROR‐induced H/R injury [54]. In contrast, lncRNA HOTAIR activates NF‐*κ*B signaling and suppresses miR‐138 expression [55]. Upregulation of miR‐138, in turn, inhibits NF‐*κ*B activity and reduces inflammatory proteins, conferring protection against H/R‐induced OS [56]. Furthermore, lncRNA KLF3‐AS1 suppresses miR‐138‐5p to increase SIRT1 expression, thereby attenuating cardiomyocyte apoptosis during myocardial infarction [57].

circRNAs also participate in miR‐138 regulation during I/R injury. circSAMD4A promotes apoptosis and inflammation in H/R‐treated cardiomyocytes while modulating miR‐138‐5p expression in AMI mouse hearts and H9C2 cells (44). Another important regulator is early growth response 1 (EGR1), a zinc finger transcription factor activated in the hypoxic and ischemic state [58]. Elevated EGR1 contributes to cardiac injury, whereas its inhibition reduces hypoxia‐induced damage [59] and blocks TLR4/NF‐*κ*B activation [60]. Mechanistically, circ_SMG6 silencing increases miR‐138‐5p levels, suppresses EGR1, and attenuates I/R injury via inhibition of the TLR4/TRIF pathway and neutrophil recruitment [61].

In summary, miR‐138 exerts cardioprotective effects against I/R injury through multifaceted mechanisms involving HIF‐1*α*, LCN2, LTB4R1, and noncoding RNAs. By modulating these targets, miR‐138 reduces apoptosis, OS, and inflammation, underscoring its therapeutic potential in mitigating myocardial reperfusion injury.

### 3.3. HF

HF is a complex clinical syndrome characterized by structural or functional cardiac abnormalities that impair ventricular filling or ejection capacity, leading to reduced cardiac output. This compromised pumping ability renders the heart unable to meet the body′s metabolic demands, or able to do so only under conditions of abnormally elevated ventricular filling pressures. HF represents the end‐stage manifestation of various CVDs and is typified by pathological features such as myocardial fibrosis and cardiomyocyte apoptosis [62].

Emerging studies indicate that miR‐138 plays an important role in the pathogenesis and progression of HF, potentially through several mechanisms. These mechanisms involve its participation in regulatory networks of noncoding RNAs, its influence on cardiomyocyte apoptosis mediated by signaling pathways such as LCN2 and SIRT1, and its modulation of myocardial fibrosis pathways including TGF‐*β*1/Smad, RhoC, and CTGF.

Among noncoding RNAs, lncRNA SOX2 overlapping transcript (SOX2OT) has drawn attention. SOX2OT is significantly upregulated in cardiac tissue of patients with both non‐end‐stage and end‐stage HF. Silencing SOX2OT expression has been shown to reduce myocardial injury [63]. Mechanistically, transforming growth factor‐*β*1 (TGF‐*β*1), a key profibrotic cytokine, promotes extracellular matrix deposition via activation of the Smad signaling pathway and regulation of fibroblast proliferation, differentiation, and migration [64]. Evidence shows that SOX2OT increases miR‐138‐5p expression, thereby activating the TGF‐*β*1/Smad signaling pathway and promoting myocardial fibrosis [65].

In addition, circRNAs have emerged as critical regulators of cardiovascular pathology [66]. CircUbe3a, a circRNA family member, contributes significantly to HF development. RhoC, an important signaling molecule of the Rho subfamily, acts as a molecular switch in multiple signal transduction pathways [67]. During myocardial hypertrophy, RhoC activation enhances smooth muscle actin promoter activity and drives fibroblast‐to‐myofibroblast transformation, thereby worsening fibrosis [68]. Li et al. reported that, after myocardial infarction, M2 macrophages release extracellular vesicles that deliver circUbe3a into cardiac fibroblasts (CFs). This promotes CF proliferation, migration, and differentiation into myofibroblasts, ultimately driving fibrosis. The profibrotic effects of circUbe3a are associated with RhoC signaling pathway activation, whereas miR‐138‐5p can partially reverse these effects [69].

Connective tissue growth factor (CTGF), another important downstream mediator induced by TGF‐*β*, also drives myocardial fibrosis. Interestingly, miR‐138‐5p directly suppresses CTGF expression, thereby attenuating fibrotic remodeling [70]. Taken together, these findings provide a strong theoretical foundation for the role of miR‐138 in mitigating myocardial fibrosis.

Beyond fibrosis, miR‐138 is also implicated in cardiomyocyte apoptosis and myocardial remodeling. On the one hand, upregulation of miR‐138 inhibits LCN2 expression, protecting cardiomyocytes from hypoxia‐induced apoptosis [46]. On the other hand, in the context of SIRT1 regulation, miR‐138‐5p exerts a proapoptotic effect. Specifically, lncRNA KLF3‐AS1 suppresses miR‐138‐5p, leading to SIRT1 upregulation and cardiomyocyte survival after myocardial infarction. Conversely, miR‐138‐5p overexpression downregulates SIRT1 expression and activity, activates p53 signaling, and ultimately promotes apoptosis, thereby aggravating HF progression [71].

In summary, current evidence highlights the dual and context‐dependent effects of miR‐138 in HF. While it alleviates myocardial fibrosis and protects against apoptosis in certain settings, it may also promote cardiomyocyte death via the SIRT1/p53 pathway. These findings underscore the complexity of miR‐138′s role in HF, and further studies are warranted to clarify its precise molecular mechanisms and therapeutic potential.

### 3.4. Pulmonary Hypertension

Pulmonary arterial hypertension (PAH) is a progressive disease characterized by distal pulmonary artery remodeling and elevated pulmonary vascular resistance. Pathological hallmarks include proliferation of pulmonary endothelial cells (PAECs), as well as activation, proliferation, and migration of pulmonary artery smooth muscle cells (PASMCs) and fibroblasts [72] Right ventricular hypertrophy and ultimately right ventricular failure can occur due to PAH progression. According to previous research, dysregulation of several miRNAs in the pulmonary vasculature, skeletal muscle, and right ventricle contributes to PAH development [73]. The underlying mechanisms involve regulation of PPAR*γ* [74], dysfunction of voltage‐gated potassium channels [75], and other pathways implicated in the pathological processes of PAH [76].

Among them, miR‐138‐5p has emerged as a pivotal regulator of PAH pathogenesis through several interconnected mechanisms. Elevated expression of miR‐138‐5p has been documented in lung tissues of patients with idiopathic pulmonary hypertension (IPAH) [77] and in hypoxia‐exposed PASMCs [78]. One major mechanism involves potassium channel regulation. Downregulation of K^+^ channel expression and current in PASMCs contributes to sustained pulmonary vasoconstriction and vascular remodeling in IPAH. Babicheva et al. demonstrated that miR‐138‐5p upregulation suppresses K^+^ channel expression and activity, thereby inducing vasoconstriction and pathological vascular remodeling [79].

In addition to vascular tone regulation, miR‐138 exerts direct effects on PASMC proliferation and apoptosis. Specifically, miR‐138 inhibits the two‐pore domain potassium channel TASK‐1 (KCNK3), leading to ERK1/2 pathway activation, increased Bcl‐2 expression, and reduced Caspase‐3 activity. This signaling cascade enhances PASMC proliferation and resistance to apoptosis. Conversely, exogenous elevation of miR‐138 can inhibit caspase activation and interfere with Bcl‐2 signaling, further suppressing PASMC apoptosis [80]. Importantly, inhibition of miR‐138‐5p restores KCNK3 expression in the lungs and alleviates monocrotaline‐induced pulmonary hypertension in animal models [78], highlighting its potential as a therapeutic target.

Beyond channel and apoptotic regulation, circRNAs serve as upstream modulators of miR‐138 in PAH. Li et al. reported that hsa_circWDR37_016 (circWDR37) is significantly upregulated in PAH and functions as a molecular sponge for miR‐138‐5p. By sequestering miR‐138‐5p, circWDR37 enhances PASMC proliferation, cell cycle progression, antiapoptotic signaling, and migration, thereby driving hypoxia‐induced pulmonary vascular remodeling [81]. This finding suggests that the circWDR37–miR‐138‐5p interaction represents a novel axis contributing to PAH pathology and a potential therapeutic target.

Collectively, these studies underscore the multifaceted role of miR‐138‐5p in PAH pathogenesis. Through regulation of potassium channel activity, modulation of PASMC proliferation and apoptosis, and interaction with circRNAs, miR‐138‐5p emerges as a critical driver of pulmonary vascular remodeling and right heart dysfunction.

### 3.5. Other CVDs

Diabetic cardiomyopathy (DCM), a diabetes‐specific complication, is a major contributor to mortality in diabetic patients. Its core pathological features include cardiomyocyte hypertrophy, interstitial fibrosis, and impaired coronary microvascular perfusion [82]. Studies demonstrate a robust association between DCM and altered gene expression patterns, with miRNAs playing essential roles in regulating these changes. During cardiac remodeling, aldosterone upregulates collagen gene expression in cardiomyocytes, promotes inflammation and OS, and causes vascular endothelial damage [83]. Aldosterone synthase (CYP11B2) is the rate‐limiting enzyme in aldosterone biosynthesis. Notably, downregulation of lncRNA GAS5 suppresses CYP11B2 expression by upregulating miR‐138‐5p, thereby ameliorating high glucose‐induced cardiomyocyte injury [84].

Alcoholic cardiomyopathy (ACM), a distinct form of dilated cardiomyopathy caused by chronic alcohol abuse, manifests as ventricular dilation, congestive HF, and reduced left ventricular ejection fraction [85]. Jing et al. first reported miR‐138‐5p as a potential biomarker for ACM [86]. Subsequent studies revealed that elevated miR‐138‐5p expression correlates positively with left atrial diameter, while inversely correlating with left ventricular ejection fraction, suggesting diagnostic potential for ACM [87].

Atrial fibrillation (AF), one of the most common arrhythmias, is characterized by atrial dilation, fibrosis, and electrical remodeling and can result in serious cardiovascular complications such as HF, stroke, and peripheral embolism [88]. Yan et al. reported significant upregulation of miR‐138‐5p and miR‐10b in the left atrium of patients with rheumatic mitral valve disease (RMVD) complicated with AF compared to those with sinus rhythm, suggesting their role in atrial structural and functional remodeling [89]. In contrast, Xie et al. observed reduced miR‐138‐5p expression in right atrial appendage tissues of AF patients compared to sinus rhythm controls. Interestingly, elevated miR‐138‐5p levels inhibited cardiomyocyte proliferation, whereas its knockdown enhanced cell growth, an effect associated with suppression of CYP11B2 expression [90]. These findings indicate that miR‐138‐5p exerts atrial region‐specific and context‐dependent effects in AF pathogenesis.

Viral myocarditis (VMC), an inflammatory cardiac disease that can progress to dilated cardiomyopathy and HF [91], is also linked to miR‐138. In coxsackievirus B3‐infected cardiomyocytes, lncRNA HIF1A‐AS1 activates NF‐*κ*B by downregulating miR‐138, thereby promoting cardiomyocyte apoptosis and inflammation. This highlights the antiapoptotic and anti‐inflammatory properties of miR‐138 in VMC [92].

Peripheral arterial disease (PAD) is another major cause of cardiovascular morbidity and mortality worldwide. Elevated circulating LCN2 has been associated with poor prognosis, including a higher risk of amputation or death [93]. Saenz‐Pipaon et al. demonstrated that serum miR‐138‐5p was downregulated in PAD patients, whereas LCN2 levels were increased. In a hindlimb ischemia (HLI) mouse model, miR‐138‐5p was shown to negatively regulate LCN2 in skeletal muscle, identifying a potential miR‐138/LCN2 axis in PAD pathophysiology [94].

Calcific aortic valve disease (CAVD), the most prevalent valvular heart disease, is marked by aortic valve thickening, calcification, and progressive hemodynamic dysfunction leading to HF [95–97]. Osteogenic differentiation of AVICs and calcium deposition are central mechanisms of CAVD progression, regulated in part by the Wnt/*β*‐catenin signaling pathway [98]. Activation of this pathway increases *β*‐catenin expression and transcriptional activation of RUNX2, a master regulator of osteogenic differentiation, thereby promoting calcification [99]. Yan et al. revealed that miR‐138‐5p attenuates osteogenic differentiation of human AVICs by inhibiting the RUNX2–Wnt/*β*‐catenin signaling axis, ultimately mitigating CAVD progression [100].

Taken together, miR‐138‐5p exerts multifaceted regulatory effects across diverse cardiovascular pathologies, including fibrosis, electrical remodeling, calcification, inflammation, and vascular repair. By modulating key pathways such as the CYP11B2/aldosterone axis, LCN2, Wnt/*β*‐catenin–RUNX2 cascade, and NF‐*κ*B network, miR‐138‐5p links gene expression changes to disease‐specific phenotypes. These findings not only provide mechanistic insights into CVD progression but also highlight miR‐138‐5p as a promising candidate for diagnostic and therapeutic strategies.

## 4. Translational and Therapeutic Prospects of miR‐138

### 4.1. miR‐138 as a Potential Biomarker

miRNAs are well‐established potent regulators of gene expression. A recent systematic review analyzing research from the past 5 years has formally defined the term “theranoMiRNA”—referring to miRNAs applicable for both diagnosis and therapy. This conceptual framework represents a significant innovation in understanding miRNA dysregulation in CVDs [101]. Among the most representative theranoMiRNAs in cardiovascular medicine, miR‐126 has garnered substantial attention due to its critical roles in angiogenesis, endothelial homeostasis, and vascular repair, with clinical studies confirming its diagnostic value [102]. By contrast, although clinical evidence for miR‐138 in CVDs remains limited, emerging studies highlight its potential diagnostic utility in other contexts. For instance, plasma methylated DNA of miR‐138‐5p was identified as a biomarker for early colorectal cancer detection, while Jing et al. reported significantly upregulated circulating miR‐138 in ACM, supporting its role as a biomarker in specific cardiovascular settings [86]. Thus, while promising, the diagnostic and prognostic relevance of miR‐138 in broader CVD contexts awaits further validation. To further elucidate the biological plausibility underlying its biomarker potential, Table 1 summarizes the currently identified downstream target genes and proteins regulated by miR‐138.

**Table 1:** miR‐138 and its downstream target genes or proteins.

**MiRNA**	**Target**		**Expression**	**Reference**
MiR‐138‐5p	ATG7	(autophagy related 7)	Suppression	[103]
MiR‐138‐5p	APOBEC3B	—	Promotion	[104]
miR‐138‐5p	Bag‐1	(Bcl‐2‐associated athanogene‐1)	Suppression	[105]
miR‐138‐5p	BRD4	(bromodomain‐containing protein 4)	Suppression	[15]
miR‐138‐5p	BMPR2	(bone morphogenetic protein receptor 2)	Suppression	[106]
miR‐138‐5p	CCND3	(cyclin D3)	Suppression	[107]
miR‐138‐5p	CDK6	(cyclin‐dependent kinase 6)	Suppression	[108]
miR‐138‐5p	CDK8	(cyclin‐dependent kinase 8)	Suppression	[109]
miR‐138‐5p	CD44st	—	Suppression	[110]
miR‐138‐5p	CREB1	(cAMP‐responsive element binding protein 1)	Suppression	[111]
miR‐138‐5p	CLN5	(ceroid lipofuscinosis neuronal protein 5)	Suppression	[112]
miR‐138‐5p	CPT1B	(carnitine palmitoyltransferase 1b)	Suppression	[113]
miR‐138‐5p	CCR7	(CC chemokine receptor 7)	Suppression	[114]
miR‐138‐5p	Cyclin E1	—	Suppression	[115]
miR‐138‐5p	DNMT3A	(DNA‐methyltransferase‐3a)	Suppression	[23]
miR‐138‐5p	ERCC1	(excision repair cross‐complementation group 1)	Suppression	[116]
miR‐138‐5p	ERCC4	(excision repair cross‐complementation group 4)	Suppression	[116]
miR‐138‐5p	E2F3	(E2F transcription factor 3)	Suppression	[117]
miR‐138‐5p	EIF4EBP1	(eukaryotic translation initiation factor 4E‐binding protein 1)	Suppression	[118]
miR‐138‐5p	EGR1	(early growth response factor 1)	Suppression	[61]
miR‐138‐5p	FOXK1	(forkhead box k1)	Suppression	[119]
miR‐138‐5p	FOXC1	(forkhead box C1)	Suppression	[120]
miR‐138‐5p	GPR124	(G protein‐coupled receptor124)	Suppression	[121]
miR‐138‐5p	HOXD11	(highlighted that homeobox D11)	Suppression	[122]
miR‐138‐5p	IGF2	(insulin‐like growth factor 2)	Suppression	[123]
miR‐138‐5P	KDM6B	(lysine demethylase 6B)	Suppression	[17]
miR‐138‐5p	KLF12	(Krueppel‐like factor 12)	Suppression	[124]
miR‐138‐5p	LTB4r1	(LTB4 receptor 1)	Suppression	[48]
miR‐138‐5p	LIPH	(lipase H)	Suppression	[125]
miR‐138‐5P	MACF1	(microtubule actin cross‐linking factor 1)	Suppression	[126]
miR‐138‐5p	MCU	(mitochondrial calcium uniporter)	Suppression	[127]
miR‐138‐5p	MACC1	(metastasis associated in colon cancer 1)	Suppression	[128]
miR‐138‐5p	MYH9	(myosin heavy chain 9)	Suppression	[129]
miR‐138‐5p	NLRP3		Suppression	[130]
miR‐138‐5p	NFIB	(nuclear factor I/B)	Suppression	[131]
miR‐138‐5p	P53	—	Suppression	[132]
miR‐138‐5p	PD‐L1	(programmed cell death ligand 1)	Suppression	[133]
miR‐138‐5p	PTK	(protein tyrosine kinase)	Suppression	[134]
miR‐138‐5p	PLLP	(plasmolipin)	Suppression	[135]
miR‐138‐5p	PEG10	(paternally expressed 10)	Suppression	[136]
miR‐138‐5p	PGC‐1*α*	(peroxisome proliferative activated receptor‐*γ* coactivator 1 alpha)	Suppression	[137]
miR‐138‐5p	PAX5	(paired box 5)	Suppression	[138]
miR‐138‐5p	P65	—	Suppression	[139]
miR‐138‐5p	PLAGL2	(pleomorphic adenoma gene like‐2)	Suppression	[140]
miR‐138‐5p	ROCK2	(Rho‐associated coiled‐coil‐containing protein kinases 2)	Suppression	[141]
miR‐138‐5p	RHBDD1	(rhomboid domain‐containing protein 1)	Suppression	[142]
miR‐138‐5p	RhoC	(Ras homolog gene family member C)	Suppression	[69]
miR‐138‐5p	SNIP1	(Smad nuclear interacting protein 1)	Suppression	[7]
miR‐138‐5p	SEMA4C	(semaphorin4C)	Suppression	[143]
miR‐138‐5p	SIN3A	(suppressor interacting 3a)	Suppression	[144]
miR‐138‐5p	SOX9	(SRY‐related high‐mobility‐group‐box 9)	Suppression	[145]
miR‐138‐5p	SOX13	(Ry‐related high‐mobility group box13)	Suppression	[146]
miR‐138‐5p	Survivin	—	Suppression	[147]
miR‐138‐5p	SelM	(selenoprotein M)	Suppression	[148]
miR‐138‐5p	SESN2	(Sestrin2)	Suppression	[149]
miR‐138‐5p	SDC3	(Syndecan 3)	Suppression	[150]
miR‐138‐5p	Snail1	—	Suppression	[151]
miR‐138‐5p	TERT	(telomerase reverse transcriptase)	Suppression	[152]
miR‐138‐5p	TRPC5	(transient receptor potential canonical 5)	Suppression	[153]
miR‐138‐5p	TNFAIP3	(tumor necrosis factor alpha–induced protein 3)	Suppression	[154]
miR‐138‐5p	TMEM40	(transmembrane protein 40)	Suppression	[155]
miR‐138‐5p	TRIP6	(thyroid hormone receptor interacting protein 6)	Suppression	[156]
miR‐138‐5p	TGF‐*β*1	(transforming growth factor‐beta 1)	Suppression	[65]
miR‐138‐5p	TGF‐*β*3	(transforming growth factor‐beta 3)	Suppression	[157]
miR‐138‐5p	TBL1X	(transducin beta‐like protein 1, X‐linked)	Suppression	[158]
miR‐138‐5p	WEE1	—	Suppression	[22]
miR‐138‐5p	WWC1	—	Suppression	[159]
miR‐138‐5p	YAP1	(Yes‐associated protein 1)	Suppression	[160]
miR‐138‐5p	ZFX	(zinc finger protein X‐linked)	Suppression	[161]
miR‐138	BMP2	(bone morphogenetic protein‐2)	Suppression	[162]
miR‐138	c‐Met	—	Suppression	[163]
miR‐138	CREPT	(cell cycle–related and expression‐elevated protein in tumor)	Suppression	[164]
miR‐138	CTGF	(connective tissue growth factor)	Promotion	[70]
miR‐138	CYP11B2	(aldosterone synthase)	Suppression	[84]
miR‐138	DEK	—	Suppression	[165]
miR‐138	DEC	(differentiated embryonic chondrocyte)	Suppression	[166]
miR‐138	EZB2	—	Suppression	[167]
miR‐138	EZH2	(Zeste homolog 2)	Suppression	[168]
miR‐138	EGFR	(epidermal growth factor receptor)	Suppression	[169]
miR‐138	E2F2	(E2F transcription factor 2)	Suppression	[170]
miR‐138	FAK	(focal adhesion kinase)	Suppression	[134]
miR‐138	FOXP4	(forkhead box P4)	Suppression	[171]
miR‐138	FBLN5	(fibulin‐5)	Suppression	[172]
miR‐138	GIT1	(G‐protein‐coupled receptor kinase‐interacting protein 1)	Suppression	[8]
miR‐138	GNAI2	(G protein alpha inhibiting activity polypeptide 2)	Suppression	[173]
miR‐138	HDAC4	(histone deacetylation 4)	Suppression	[174]
miR‐138	H2AX	—	Suppression	[175]
miR‐138	HIF‐1*α*	(hypoxia‐inducible factor‐1 alpha)	Suppression	[43]
miR‐138	ICP0	—	Suppression	[176]
miR‐138	ISG15	(interferon‐stimulated gene 15)	Suppression	[177]
miR‐138	IGF2BP2	(insulin‐like growth factor 2 mRNA binding protein 2)	Suppression	[178]
miR‐138	KDM2A	(lysine demethylase 2A)	Suppression	[179]
miR‐138	KDR	(kinase insert domain receptor)	Suppression	[180]
miR‐138	K2	(Kindlin‐2K)	Suppression	[181]
miR‐138	LIMK1	(LIM kinase 1)	Suppression	[182]
miR‐138	LCN2	(Lipocalin 2)	Suppression	[46]
miR‐138	Mst1	(mammalian sterile 20‐like kinase 1)	Suppression	[55]
miR‐138	NF‐*κ*B	(NF‐kappaB)	Suppression	[56]
miR‐138	OX40L	—	Suppression	[183]
miR‐138	PDK1	(3‐phosphoinositide‐dependent kinase 1)	Suppression	[184]
miR‐138	PPAR*β*	(peroxisome proliferator–activated receptor beta)	Suppression	[185]
miR‐138	PKM2	(pyruvate kinase M2)	Suppression	[186]
miR‐138	PI3K	(phosphatidylinositol 3‐kinase)	Suppression	[31]
miR‐138	REIN	(reelin)	Suppression	[187]
miR‐138	RUNX3	—	Suppression	[188]
miR‐138	RMND5A	(required for meiotic nuclear division 5 homolog A)	Suppression	[185]
miR‐138	ROBO4	(Roundabout 4)	Suppression	[189]
miR‐138	RARA	(retinoic acid receptor alpha)	Suppression	[190]
miR‐138	SIRT1	(Sirtuin 1)	Suppression	[38]
miR‐138	SIRT2	(Sirtuin 2)	Suppression	[191]
miR‐138	SIRT6	(Sirtuin 6)	Suppression	[192]
miR‐138	SOX4	(SRY‐related high‐mobility‐group box 4)	Suppression	[193]
miR‐138	SOX12	(SRY‐related high‐mobility‐group box 12)	Suppression	[194]
miR‐138	SGTA	(small glutamine‐rich TPR‐containing protein A)	Suppression	[195]
miR‐138	SP1	(specificity protein 1)	Suppression	[196]
miR‐138	S100A1	—	Suppression	[28]
miR‐138	THBS1	(Thrombospondin‐1)	Suppression	[197]
miR‐138	TWIST2	(twist basic helix‐loop‐helix transcription factor 2)	Suppression	[198]
miR‐138	TUSC2	(tumor suppressor candidate 2)	Suppression	[199]
miR‐138	TCF3	(transcription factor 3)	Suppression	[200]
miR‐138	TLR3	(Toll‐like receptor 3)	Suppression	[201]
miR‐138	TASK‐1	(Twik‐related acid‐sensitive potassium channel‐1)	Suppression	[80]
miR‐138	VIM	(vimentin)	Suppression	[202]
miR‐138	USP10	—	Suppression	[203]
miR‐138‐1‐3p	CRIPTO	—	Suppression	[204]
miR‐138‐1‐3p	PAK5	(p21‐activated kinase 5)	Suppression	[205]

### 4.2. Therapeutic Applications of miR‐138

Therapeutic exploration of miR‐138 largely focuses on its regulatory networks and interaction with pharmacological agents (Table 2). Investigations into traditional Chinese medicine (TCM) mechanisms reveal encouraging prospects. For example, SYB downregulates miR‐138, elevates S100A1, and enhances eNOS activity, thereby mitigating endothelial injury [35]. Similarly, hyperoside (Hyp) confers cardioprotection by upregulating miR‐138, suppressing its downstream targets MLK3 and LCN2, and reducing hypoxia‐induced apoptosis [207]. Resveratrol (RSV), with pleiotropic cardiovascular benefits, also modulates miR‐138 levels, increasing FAK expression, promoting endothelial progenitor cell (EPC) migration, and facilitating thrombus recanalization in vivo [208–210]. Collectively, these findings underscore the therapeutic promise of miR‐138 modulation in ischemic heart disease and HF.

**Table 2:** miR‐138 and its function in the cardiovascular system.

**CVD**	**Subjects**	**Mechanism**	**Effect**	**Function**	**Reference**
AS	ECs, HMVEC	miR‐138/S100A1/eNOS	Suppression	Reduces nitric oxide (NO) production, contributing to endothelial dysfunction.	[28]
	Diabetic VSMCs	miR‐138/SIRT1	Suppression	Promotes VSMC proliferation and migration, and enhances pro‐inflammatory cytokine expression (e.g., IL‐6 and IL‐1*β*).	[38]
	OX‐LDL‐induced HCAECs	miR‐138/PI3K/Akt/eNOS	Suppression	Reduces inflammatory factor expression (e.g., TNF‐*α*, IL‐6, and IL‐8) and alleviates HCAEC injury and inflammation.	[31]
	OX‐LDL‐induced HUVECs、Western diet‐induced ApoE−/− mouse	miR‐138/APT1/H‐Ras/MAPK	Suppression	Reduces expression of MMP, ICAM‐1, and VCAM‐1.	[11]
I/R injury	Mouse model of myocardial I/R injury, myocardial H9C2 cells	miR‐138/HIF‐1*α*	Suppression	Inhibits Caspase‐9 and Caspase‐3 expression, and reduces cardiomyocyte apoptosis.	[43, 44]
	Hypoxia‐induced HL‐1 cells	miR‐138/LCN2	Suppression	Inhibits hypoxia‐induced cardiomyocyte apoptosis.	[46]
	Mouse model of myocardial I/R Injury	miR‐138‐5p/Ltb4r1	Suppression	Reduces inflammatory factor and collagen fiber expression, and alleviates myocardial injury.	[48]
	H/R‐induced injury of H9C2 cells	lncRNA ROR/miR‐138/Mst1	Suppression	Reduces H/R‐induced myocardial cell injury.	[55]
	H/R‐induced injury of H9C2 cells	lncRNAHOTAIR/miR‐138/NF‐*κ*B	Suppression	Reduces H/R‐induced myocardial cell damage and inflammatory response.	[56]
	Mouse model of myocardial I/R Injury、H/R‐induced injury of H9C2 cells	lncRNA KLF3‐AS1/miR‐138/SIRT1	Suppression	Reduces H/R‐induced myocardial cell damage and inflammatory response.	[57]
	Mouse model of myocardial I/R Injury、H/R‐induced injury of H9C2 cells	circSAMD4A/miR‐138‐5p	—	Reduces H/R‐induced myocardial cell damage and inflammatory response.	[206]
	Mouse model of myocardial I/R injury	circ_SMG6/miR‐138‐5p/EGR1/TLR4/TRIF	Suppression	Reduces neutrophil recruitment and myocardial injury.	[61]
HF	HF mouse, ISO‐induced CFs	lncRNASOX2/miR‐138‐5P/TGF‐*β*1/smad	Suppression	Improves myocardial fibrosis.	[65]
	Mouse CFs, MI mouse	CircUbe3a/miR‐138‐5p/RhoC	Suppression	Improves myocardial fibrosis.	[69]
	H/R‐induced injury of H9C2 cells	miR‐138/CTGF	Promotion	Improves myocardial fibrosis.	[70]
	Hypoxia‐induced HL‐1 cells	miR‐138/Lcn2	Suppression	Inhibits myocardial cell apoptosis.	[46]
	MI mouse, H/R‐induced injury of H9C2 cells	lncRNA KLF3‐AS1/miR‐138/SIRT1	Suppression	Aggravates cardiomyocyte apoptosis.	[57]
	HF models、and HCM cells	miR‐138‐5P/SIRT1/p53	Suppression	Aggravates cardiomyocyte apoptosis.	[71]
PAH	hPASMCs	miR‐138/KCNK3/ERK1/2/Bcl‐2/Caspase‐3	Suppression	Inhibits PASMC proliferation, and attenuates vascular remodeling and pulmonary vasoconstriction.	[80]
	HPASMCs	circWDR37/miR‐138	—	Inhibits PASMC proliferation, and attenuates vascular remodeling and pulmonary vasoconstriction.	[81]
DCM	HG‐induced AC16 cardiomyocytes and STZ‐induced rat diabetes model	lncRNAGAS5/miR‐138/CYP11B2	Suppression	Reverses HG‐induced myocardial cell damage.	[84]
ACM	—	—	—	A biomarker of ACM.	[86]
AF	AC16 cells	miR‐138‐5P/CYP11B2	Suppression	Inhibits cardiomyocyte proliferation.	[90]
VMC	CVB3‐induced cardiomyocytes	lncRNAHIF1A‐AS1/miR‐138/NF‐*κ*B	Suppression	Reduces myocardial cell apoptosis and inflammatory response.	[92]
PAD	HL‐I mouse	miR‐138/LCN2	Suppression	Inhibits the anti‐inflammatory response of LCN2	[94]
CAVD	Human aortic valve tissue, hAVICs	miR‐138‐5p/RUNX2/Wnt/*β*‐catenin	Suppression	Reduces myocardial cell apoptosis and inflammatory response.	[100]

Abbreviations: ApoE−/−, apolipoprotein E‐deficient; CFs, cardiac fibroblasts; ECs, endothelial cells; HCAECs, human coronary artery endothelial cells; HG, high glucose; HMVEC, human microvascular endothelial cells; hPASMCs, human pulmonary arterial smooth muscle cells; HUVEC, human umbilical vein endothelial cells; ISO, isoprenaline; MI, myocardial infarction; OX‐LDL, oxidized low‐density lipoprotein; STZ, streptozotocin; VSMCs, vascular smooth muscle cells.

### 4.3. miR‐138 and Stent‐Based Therapies

miRNA‐targeted approaches have also been investigated in stent development, particularly for preventing in‐stent restenosis (ISR). For example, miR‐125 has been incorporated into drug‐eluting stents (DES) to reduce ISR [211], while knockdown of miR‐21 and miR‐221 decreases neointimal formation after vascular injury [212]. Given miR‐138′s regulatory roles in angiogenesis, vascular inflammation, and remodeling, it represents a promising candidate for next‐generation DES technologies aimed at reducing restenosis risk.

### 4.4. Delivery Strategies and Translational Challenges

In cardiovascular medicine, miRNA‐based theranostic strategies are increasingly supported by advances in RNA delivery platforms, including engineered viral vectors, optimized lipid nanoparticles (LNPs), and exosome‐based systems, all of which improve delivery efficiency and tissue specificity. Nevertheless, practical barriers persist, such as optimizing administration routes, maintaining in vivo stability, and ensuring cell type–specific targeting [213]. Current experimental approaches to miR‐138 delivery include small cell–penetrating molecules [214], miRNA–drug combination therapies [215], miRNA–siRNA conjugates [216], artificially engineered constructs [217], and miRNA sponges [218]. Although these strategies remain preclinical, they provide a foundation for future translational applications.

### 4.5. Summary and Outlook

In summary, although direct clinical validation of miR‐138 in CVDs is currently lacking, preclinical findings strongly support its potential as both a biomarker and therapeutic target. Beyond its diagnostic implications, pharmacological modulation of miR‐138 and integration into stent‐based therapies offer novel therapeutic opportunities. Continued refinement of delivery systems and validation in human studies will be critical for realizing miR‐138′s translational promise in CVD management.

## 5. Conclusion

This review systematically elucidates the central regulatory role of miR‐138 in CVDs, including AS, ischemia–reperfusion injury, HF, and pulmonary hypertension. By targeting key molecules and pathways—such as S100A1/eNOS, SIRT1, HIF‐1*α*, LCN2, LTB4r1, TGF‐*β*/Smad, RhoC, and KCNK3—miR‐138 demonstrates significant bidirectional (disease‐promoting/protective) and pleiotropic characteristics, establishing its molecular foundation as a “theranoMiRNA” with integrated diagnostic and therapeutic potential.

## 6. Future Directions

Despite the substantial progress summarized in this review, several limitations currently constrain the translational potential of miR‐138. First, most existing evidence derives from preclinical models, while clinical validation in human patients remains sparse, particularly in cardiovascular contexts. Second, the spatiotemporal and cell type–specific expression patterns of miR‐138 have not been fully mapped, leaving critical uncertainties about its functional heterogeneity. Third, the mechanistic plasticity of miR‐138—switching between protective and pathogenic roles depending on context—remains poorly understood, complicating therapeutic applications. Finally, challenges in delivery efficiency, tissue specificity, and long‐term safety continue to hinder clinical translation of miRNA‐based therapies.

Future research should therefore prioritize the following: (1) applying spatial transcriptomics and single‐cell sequencing to define tissue‐ and disease‐specific expression networks of miR‐138; (2) integrating conditional gene models with AI‐driven multiomics to resolve molecular switching mechanisms, with a particular focus on ceRNA networks involving lncRNAs and circRNAs; (3) conducting large‐scale, prospective, multicenter cohort studies to validate circulating miR‐138‐5p and its methylated forms as standardized diagnostic and prognostic biomarkers; and (4) exploring synergistic strategies combining miR‐138 modulators with conventional cardiovascular drugs (e.g., statins and SGLT2 inhibitors) or other nucleic acid therapies (e.g., siRNAs), thereby maximizing therapeutic efficacy while minimizing resistance.

Only through addressing these limitations with systematic, well‐funded investigations can the full translational potential of miR‐138 as a next‐generation theranoMiRNA in cardiovascular medicine be realized.

## Conflicts of Interest

The authors declare no conflicts of interest.

## Funding

No funding was received for this manuscript.

## Data Availability

Data sharing is not applicable to this article as no new data were created or analyzed in this study.
